# U-Shaped Relationship between Years of Residence and Negative Mental Health Outcomes among Rural-to-Urban Children in Migrant Schools in Beijing, China: The Moderating Effects of Socioeconomic Factors

**DOI:** 10.3389/fpubh.2017.00168

**Published:** 2017-08-02

**Authors:** Jin Cheng, Ri-chu Wang, Xing Yin, Lin Fu, Zheng-kui Liu

**Affiliations:** ^1^CAS Key Laboratory of Mental Health, Institute of Psychology, Chinese Academy of Sciences, Beijing, China; ^2^University of Chinese Academy of Sciences, Beijing, China

**Keywords:** migrant children, length of residence, quadratic relationship, anxiety, depression

## Abstract

**Aim:**

This study aimed to test the relationship between length of residence and mental health in a school-based sample of migrant children who studied in migrant schools.

**Methods:**

A total of 7,296 rural-to-urban migrant children were recruited from 58 schools in Beijing and assessed by the State-Trait Anxiety Inventory and Children’s Depression Inventory.

**Results:**

A quadratic relationship was found between mental health and length of residence. The results suggested that the scores for anxiety and depression were high during the initial resettlement after migrating and then decreased. However, after approximately 8 years, the scores increased. Our findings also showed a significant moderating effect of family socioeconomic status on the relation between mental health and length of residence.

**Conclusion:**

This study provided empirical evidence for a better understanding of psychosocial factors on the mental health of migrant children during the process of urbanization in China.

There has been a considerable amount of population flow into cities in the process of urbanization in China. According to newly released statistics, the number of rural-to-urban migrants reached about 230 million in 2012, which comprised 17% of China’s total population. Rather than leave their youngsters behind, many migrant parents take their children to cities. Approximately 20.8% of China’s rural-to-urban migrants are school-aged children (1). Migrant children are defined by the 2005 Chinese Population Consensus as children and teenagers from 7 to 15 years of age who have lived in the inflow areas over half a year with their parents or guardians (2). The *hukou* system (China’s household registration system) casts institutional exclusion over rural-born citizens, children included. *Hukou* is the most basic and essential personal identification in China. The differences between rural and city are significant, with urban *hukou* (especially those of big cities, such as Beijing) enjoy much more rights than rural *hukou*. One’s birth *hukou* is determined by his/her birth place and *hukou* of parents. Although *hukou* can be changed (such as ownership of real estate and employment by state-owned enterprises and institutions), most migrants are incapable to do so. Many migrants cannot receive the same rights as the urban citizens; thus, their children are denied (or limited) access to education, medication, social welfare, and other social services. *Hukou* system also cast stress on rural-to-city migrants and cause discrimination against them (3). Migrant children show more negative self-perception and lower self-esteem than their native urban counterparts (1, 4, 5). Compared to city children, migrant children witness much more violence and gambling in their community (6) and experience more negative life events (7). All these factors increase the risks of externalizing and internalizing problems for migrant children. Existing studies on Chinese migrant children have indicated that migrant children are more emotionally vulnerable, depressed, and lonely, and exhibit more externalizing and internalizing of symptoms, and thus, their psychological health is in poor condition (8, 9).

For migrants, the process of adjustment to the new environment can be difficult and complex and it takes time to overcome practical and psychological difficulties and rebuild their lives. Length of residence in the new environment is closely related to the adjustment and acculturation process (10). Berry et al. (11) proposed a liner relationship between the length of residence and adaptation: the longer the duration of residence in the novel environment, the greater the application of an integration strategy, and the more experience for migrants of positive outcomes (11). However, the findings are not consistent. Markovizky and Samid (12) found that the first year after immigration was associated with poorer physical and psychological health and followed by a rise in acculturation in the second year after new immigrants from the former Soviet Union moved to Israel (12). Ward et al. (13) traced the changes in the depression of 35 Japanese students who studied in New Zealand for 1 year since arrival. He measured their depression levels at four different time points (24 h, 4 months, 6 months, and 12 months after migrating) and found that all participants tended to have a high level of depression at first, which declined remarkably but remained nearly stable in the following 4, 6, and 12 months.

Three important problems with the aforementioned studies may limit the applicability of the results to long-term effect on intranational migrant children. First, these studies only focus on the first few years after migration. Rather than tracking comparatively small numbers of participants since their arrival, some researchers have used cross-sectional methodology to investigate the effect of length of residency on migrants’ mental health in a larger community sample. Hwang et al. (14) found that longer length of residence was associated with less risk of experiencing depressive episodes among 1,724 Chinese-Americans. However, others could not replicate this liner relationship. For instance, Oppedal et al. (15) found that psychological adaptation did not change significantly as a function of length of residence in the new environment.

Second, most studies on migrants’ mental health have dealt with adult international migrants, i.e., people who move from their country of origin to a new country. It has been suggested that migrants from less developed countries are generally in poorer mental health compared to their counterparts in the host countries (16). Thus, it may be inappropriate to apply the evidence from international migrant populations to understanding intra-national migrants, especially among young population. Thus, studies on the long-term effects of intra-national migration are warranted. There is little previous literature concerning the relation between mental health and length of residence among school-aged internal migrants in China, and the few studies that have been done produced somewhat inconsistent findings. Some suggested that the length of residence did not have a significant effect on psychological adjustment in rural-to-urban migrant children, while others argued that longer urban residence was associated with a reduced risk of mental health for those who had migrated from townships or smaller cities (3). Thus, to understand the unique experience of rural-to-urban migrant children, further research is needed.

Third, previous studies generally consider acculturation as the primary contributor to mental health. In fact, difficulty in acculturation only partly contributes to migrants’ mental health risk (17, 18). It has been argued that the benefit of long-term residency may be influenced by the neighborhood characteristics (19). In a recent Chinese study, researchers found that the disadvantage in psychological health and behavioral problems disappeared after controlling for family and school characteristics (4). Thus, the investigation of a potential link between length of residence and mental health requires consideration of family socioeconomic factors. Mental health may be influenced by one’s socioeconomic status (SES) directly and indirectly. Experience of economic disadvantage during early childhood has various negative effects on children’s behavioral, cognitive, and emotional development (20). Lower SES is associated with the lack of social support, negative family circumstances (e.g., negative parenting, family conflicts, and standard of living), and poor psychological resources (e.g., self-esteem, meaningfulness, and locus of control), which in turn influence one’s mental health (21, 22). A very large proportion of migrant children began to come into urban areas from rural areas with low family SES, and they lived in crowded, chaotic, and poor housing conditions (6), known as an “urban village.” The limited education of migrant adult may influence the mental health of their children. For instance, adolescents whose parents had less than a college degree had 1.2–1.5 times higher odds of mental disorder (23). Because of a relatively disadvantageous situation economically, each parent was busy working which resulted in less communication with their children and led to a poorer quality parent-child relationships (24). That may adversely affect parent–child relationship quality, which in turn contributes to fewer psychological resources (21). The association between mental health and length of residence may vary according to the family’s SES, which has important implications for policy, but this has yet to be studied.

## The Present Study

The present study examines the relationships among length of residence, family SES, and mental health, utilizing data drawn from a large sample of rural-to-urban migrant children in Beijing, China. On the basis of previous research, we assumed an inverted U-shaped relationship between length of residence and negative mental health outcomes, namely anxiety and depression (i.e., first increase then decrease). Subsequently, we examined to see if the association between length of residence and mental health was moderated by family socioeconomic levels. Migrant children comprise 23.83% of the total population of children in Beijing, which is the highest ratio of migrant children to all children among all big cities in China (2). Because of the *hukou* system, rural-to-urban students without *hukou* of the host city are unlikely to be accepted in public education system. As a solution, civilian-run schools specifically for rural migrant children have been set up. There were 157 such schools registered with the Beijing Community Administration Bureau in 2012, with about 100,000 students (25). Unlike expensive private schools, migrant schools have very poor condition and little resources and children in such schools usually come from blue-collar families. Compared to migrant children in public schools, where migrant children are surrounded by urban peers, migrant children in migrant schools have much less opportunities to interact with urban children and reported more mental problems (26). Thus, we focused on children in migrant schools in the present study.

## Methods

### Sample

The sample for the present study consisted of 7,296 rural-to-urban migrant children (4,228 boys and 3,010 girls; 58 did not report gender) of grades 4–9 from 58 migrant schools in Beijing. The average age, adjusted for continuity of the participants, was 11.37 years (SD = 1.77). The average length of residence in Beijing was 4.52 years (SD = 3.31). Table 1 presents the demographic and sociological characteristics of the sample.

**Table 1:** Characteristics of the sample (*N* = 7,296).

Demographic variables	*M* (SD)/percentage
Gender	
Boy	57.9
Girl	41.3
Unknown	0.8
Grade	
Fourth	29.1
Fifth	28.4
Sixth	25.2
Seventh	7.1
Eighth	7.1
Ninth	3.2
Age, years	11.37 (1.77)
Parents’ relationship	
Very good	55.9
Good	22.8
Neither good nor bad	15.2
Bad	3.5
Very bad	1.8
Unknown	0.8
SES	8.56 (1.8)
Length of residence, years	4.52 (3.31)

*Percentage is given for categorical variables; *M* (SD) is given for continuous variables*.

### Procedure

The survey of the present study was a part of a large program aiming at evaluating academic achievements of migrant children. Migrant schools were contacted by the education administrative departments of each district. For this particular study, there were two basic roles in selecting migrant schools: (1) had registered to Beijing Municipal Commission of Education and (2) had more than 300 students. At least one school was selected from each of the 16 districts of Beijing. If there were no migrant schools having more than 300 students in a district, the school with the largest number of students was selected for the study. A total number of 58 schools agreed to take part in the study. All students who attended school on the evaluation day participated in the current study, with expectation of preschool and grades one to three students who were incapable to read or understand the questionnaires. Permissions were obtained from the school administrators and young participants after they were clearly informed of the purpose. Data were collected between May and July 2010. A survey of child and adolescent life was administered with the assistance of class advisers during school time, over a session of 45 min. The examiners were postgraduates majoring in psychology with formal investigation training. During the survey, examiners explained every questionnaire and students could ask examiners questions that they did not understand or it is unclear about questionnaires at any time. The study design and procedure were proved by the Ethics Review Committee of the authors’ institute.

### Measures

#### Self-Reported Depression

The Children’s Depression Inventory is a widely used self-rating scale for measuring depression in children and adolescents 7–17 years of age (27). It consists of 27 items regarding cognitive, emotional, and behavioral aspects of depressive symptoms. For each item, participants are asked to choose one of the three statements that best describes them for the past 2 weeks. The scores of individual items are combined as an indicator of self-reported level of depression, with a higher score reflecting greater depression. For the current sample, the coefficient alpha value was 0.85.

#### Self-Reported Anxiety

The State-Trait Anxiety Inventory (STAI) consists of 40 items, which have been used to measure anxiety related to anxiety tension, nervousness, and worry (28). The scale is composed of two subscales to assess state anxiety (temporary condition of anxiety) and trait anxiety (general and long standing feelings of anxiety). Each subscale consists of 20 items that are rated on a 4-point scale ranging from *almost never* to *almost always*, and the sum score of each subscale indicates self-reported level of anxiety for either the current state or the general pattern, with higher scores indicating higher levels of anxiety. Cao and Liu had revised the STAI among Chinese children and adolescents (29, 30). The combined Cronbach’s α coefficient was .80 for the State Anxiety subscale and .81 for the trait anxiety subscale.

#### Length of Residency

One question of “how long have you lived in Beijing?” were asked to measure the length of residency.

#### Family SES

The measurements of SES of children usually include occupation of the parents, education level of parents, and family income/financial resources. Since many children did not know the exact occupation of their parents and family income, we used a combination of parents’ education level and perceived family financial status as an indicator of SES in the present study. Parents’ education level was divided into five categories (1 = *below primary school*, 2 = *primary school*, 3 = *middle school*, 4 = *high school*, and 5 = *college or above*). The sum of the two items was used as an indicator of family SES, with a higher score meaning a higher SES level. For the financial status, Participants were asked to rate “How would you rank your family’s economical level in your locality?” on a 5-point scale, ranging from 1 = *lowest* to 5 = *highest*.

### Analytic Strategy

Analyses were performed by SPSS 20.0, and all figures were drawn by Excel 2007 according the outcome.

Polynomial regression analyses were conducted to test the hypothesized U-shaped relationship between the length of residence and mental health. The equation is

(1) $$y=\beta_{0}+\beta_{1}x_{1}+\beta_{2}x_{1}^{2}+\varepsilon_{0},$$

where the outcome (*y*) is:

(2) $$\begin{array}{l} (y)=\beta_{0}+\beta_{1}x_{1}\text{(Length of residence) }+\;\beta_{2}x_{1}^{2}+\text{Control variables} \end{array}$$

As such, psychological adjustment, anxiety, and depression were separately used as outcome factors and predicted by the length of residence, quadratic term of length of residence, and control variables.

The turning point occurs at the following value of the predictor length of residence (31):

(3) $$X_{i}=-\beta_{1}\;/\;2\beta_{2}$$

The turning point depends on the values of β_1_, the regression coefficient of length of residence, and β_2_, the regression coefficient of the quadratic term of the length of residence.

A hierarchical regression was used to examine the moderator effect of socioeconomics on the relationship between length of residence and mental health. We entered the variables into the regression model in four steps: (1) SES; (2) SES and length of residence; (3) SES, length of residence, and squared length of residence; and (4) SES, length of residence, squared length of residence, SES × length of residence, and SES × squared length of residence. The moderator effect can be verified from Step 4 by the test of significance of regression coefficients of the SES × length of residence and SES × squared length of residence.

## Results

The mean scores for self-reported depression, state anxiety and trait anxiety were 40.33 (SD = 7.64, range = 27–81), 39.94 (SD = 9.46, range = 20–80), and 43.50 (SD = 8.64, range = 20–79) in this sample, respectively.

**Figure 1:**
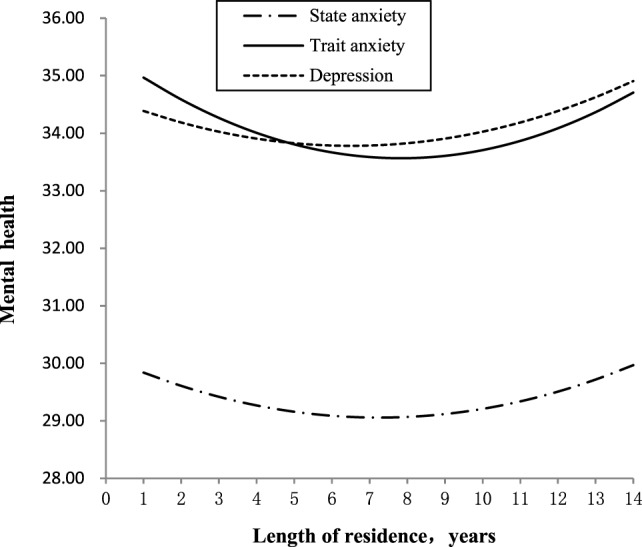
The quadratic relationship between length of residence and mental health.

Table 2 presents the results of the polynomial regression analysis examining the relationship between psychological adjustment and length of residence. The results showed a significant negative relationship between length of residence and anxiety as well as depression. The quadratic effects of length of residence predicting trait anxiety and depression were statistically significant positively but not significant for predicting state anxiety, suggesting the relationships between length of residence, and both trait anxiety and depression resemble a U-shaped configuration (Figure 1). Using formula (2) mentioned earlier, we found that the turning point was 7.44 for trait anxiety and 8.00 for depression. This means that an increasing length of residence will initially tend to decrease the levels of trait anxiety and depression, but the levels tend to increase after living in Beijing for 8 years. Girls reported less depression than boys. The levels of psychological adjustment decreased with the advancement of school grade.

**Table 2:** Quadratic relationship between length of residence and mental health.

Variable	Psychological adjustment											
	State anxiety				Trait anxiety				Depression			
	*B*	SE	β	*t*	*B*	SE	β	*t*	*B*	SE	β	*t*
Gender (1 = boy, 2 = girl)	−0.19	0.24	−0.01	−0.85	0.43	0.22	0.02	1.94	−0.65	0.18	−0.42	−3.61***
Grade	1.14	0.09	0.17	13.06***	0.71	0.08	0.11	8.88***	0.62	0.07	0.11	9.53***
Length of residence	−0.29	0.12	−0.10	−2.29*	−0.47	0.11	−0.18	−4.38***	−0.26	0.09	−0.11	−2.81**
Squared length of residence	0.02	0.01	0.07	1.61	0.03	0.01	0.14	3.28***	0.02	0.01	0.08	2.06*
			
	*R*^2^ = 0.099				*R*^2^ = 0.070				*R*^2^ = 0.097			

*p < 0.05.

**p < 0.01.

***p < 0.001.

Table 3 presents the results of analyses of hierarchical regression examining how SES moderated the relationship between length of residence and mental health. There were significant correlations between the main effect of length of residence and state anxiety, trait anxiety and depression in Step 4. A significant quadratic effect of length of residence predicts mental health. The squared length of residence was positive in Step 4 of the models correlated with state anxiety, trait anxiety, and depression. The interaction effect between socioeconomics and length of residence in Step 4 of the models was statistically negatively correlated with state anxiety and trait anxiety, but there was no significant correlation with depression.

**Table 3:** Relationship between length of residence mental health as moderated by SES.

Predictor	Mental health								
	State anxiety			Trait anxiety			Depression		
	*B*	β	*R^2^* (Δ*R^2^*)	*B*	β	*R^2^* (Δ*R^2^*)	*B*	β	*R^2^* (Δ*R^2^*)
Step 1									
SES	−0.69***	−0.13***	0.001*** (0.001***)	−0.59***	−0.12***	0.001** (0.001**)	−0.56***	−0.13***	0.001*** (0.001***)
Step 2									
LOR	−0.04	0.01	0.016***(0.017***)	−0.10**	−0.04**	0.015*** (0.014***)	−0.06	−0.03	0.018*** (0.017)
SES	−0.69***	−0.13***		−0.59***	−0.12***		−0.56***	−0.13***	
Step 3									
LOR	−0.40**	−0.14**	0.018*** (0.012**)	−0.59***	−0.22***	0.019*** (0.003***)	−0.30***	−0.13***	0.019*** (0.001**)
SES	−0.68***	−0.13***		−0.59***	−0.12***		−0.55***	−0.13***	
Squared LOR	0.03**	0.13**		0.05***	0.19***		0.02**	0.11**	
Step 4									
LOR	−0.41**	−0.14**	0.020*** (0.002**)	−0.59***	−0.22***	0.021*** (0.012**)	−0.31**	−0.13**	0.020*** (0.001)
SES	−0.61***	0.09***		−0.52***	−0.11***		−0.50***	−0.12***	
Squared LOR	0.03**	0.13**		0.05***	0.19***		0.02**	0.12**	
SES × LOR	−0.18*	−0.11*		−0.16*	−0.11*		−0.09	−0.07	
SES × squared LOR	0.01	0.08		0.01	0.07		0.01	0.07	

*p < 0.05.

**p < 0.01.

***p < 0.001.

*N = 4,898–5,615*.

*SES, socioeconomic status; LOR, length of residence; B, regression coefficient; β, beta coefficient, R^2^, significant level*.

**Figure 2:**
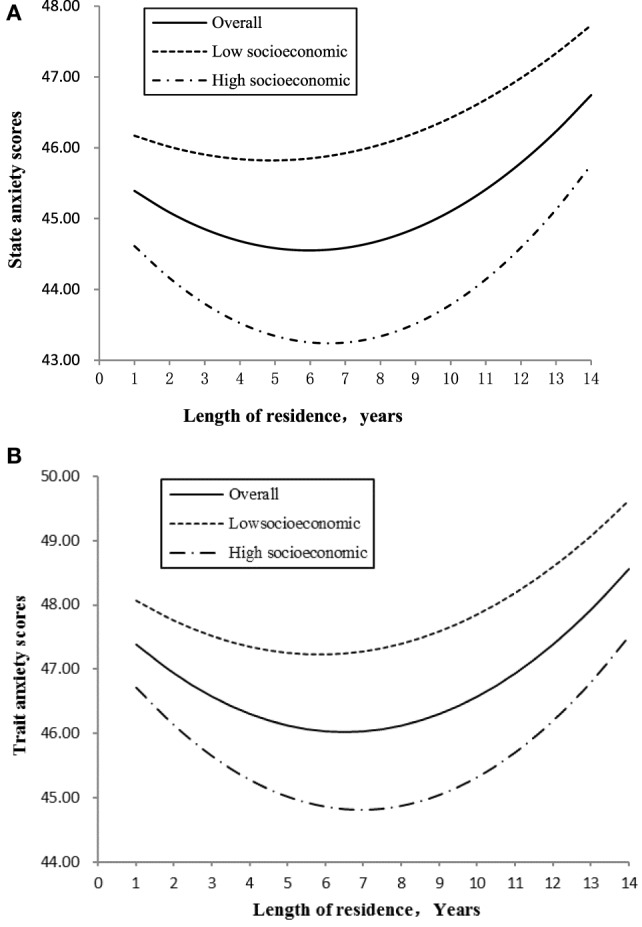
**(A)** The moderating effect of family socioeconomic status (SES) on the relationship between length of residence and state anxiety. **(B)** The moderating effect of family socioeconomic status on the relationship between length of residence and trait anxiety.

A further examination of the moderate effect of SES on state and trait anxieties, we compared high SES group (SES scored 1 SD above mean) and low SES group (SES scored 1 SD below mean). Figure 2A,B illustrates the SES moderated effects of length of residence on state anxiety and trait anxiety. Low SES group showed significant higher score on both state and trait anxieties at any time. In addition, the shapes of the curve are different between high and low SES groups for trait anxiety. Table 4 presents the regression coefficients and turning points. The results showed that the quadratic relationship between length of residence and mental health was moderated by SES: in the first few years after rural-to-urban migration, the level of anxiety began decreasing more rapidly for high SES than that for low SES. The turning point in terms of length of residence means that the change from a decrease in anxiety was much later for high SES than for low SES.

**Table 4:** Moderating effects of socioeconomic status on the quadratic relationships between length of residence and mental health.

Mental health	Regression coefficients (*B*)			*X_i_*
	Intercept	Linear	Quadratic	
State anxiety				
Low socioeconomic (−1.00 SD)	46.37	−0.23	0.02	5.75
High socioeconomic (+1.00 SD)	45.15	−0.59	0.05	5.90
Trait anxiety				
Low socioeconomic (−1.00 SD)	48.45	−0.42	0.04	5.25
High socioeconomic (+1.00 SD)	47.41	−0.75	0.05	7.50

## Discussion

Findings from the current research illustrated a quadratic U-shaped relationship between length of residence and negative mental health outcome (depression and anxiety) among rural-to-urban migrant children in Beijing. Surprisingly, the relationship is U-shaped but not inverted U-shaped as we assumed. All existing hypotheses suggested that mental health get better, or at least not getting worse when migrants lives long: a liner relationship (the longer the better) by Berry et al. (11), a U-shaped relationship (first decline then improve) by Markovizky and Samid (12), or a flat relationship (staying relatively unchanged in later years) by Oppedal et al. (15). This result is on the opposite side. However, the result of the present study suggested that self-reported anxiety and depression among students in migrant school first declined then kept increasing for years after they moved from rural country to Beijing.

The difference in the results may partially due to the different developmental stages of the subject. The results of previous research were found in adult samples, however, and the acculturation process is considered different for adolescents (32). Previous reports have shown that adolescents had a faster pace of psychological adjustment compared to adult immigrants (33). The later increase in anxiety and depression may reflect a general pattern of increased depression and anxiety during adolescence (34). In addition, we consider that there would be an adaptive process. In a new city, children know surroundings and meet new friends at early to adapt. After this stage, migrant children also face migration-specific assimilation stress which has an impact on their psychological health (35). Based on Erikson’s stage theory of psychosocial development, Erikson (36) identities that crisis is the core reason for psychological problems in adolescents. “Searching for who I am” is the fundamental developmental task for teenagers. For Chinese rural-to-urban migrant children, this crisis is even worse because of the *hukou* system, which institutionally separates rural migrants from regular urban citizens (3, 35). In the identity stage, the migrant children would feel their difference and discrimination about their cultural identity from the locals. Therefore, the levels of their anxiety and depression increased after several years.

Prior studies have suggested that with increasing duration in Beijing, migrant children display increased willingness to integrate into the city on one hand while experiencing a stronger sense of exclusion on the other hand, the conflicts between the two facets resulted in anxiety and depression (37). Since all participants in the current study were students in migrant schools, which are outcast of urban education system. The stereotype and inferior perception associated with the school may amplify the psychological conflict in migrant children.

In this study, the results showed that lower SES was associated with higher anxiety and depression, which is consistent with a number of previous studies, which suggest that parents’ SES is positively related to social and cultural adaptation, and the self-esteem of migrant children, but negatively related to loneliness and depression [e.g., Ref. (3, 35, 38)]. This further illustrates the importance of socioeconomics, and also how the pattern of relationships between length of residence and mental health varied for different levels of socioeconomics, namely, how a high level of family SES will benefit children for the process of acculturation after migration (39).

It is also worth noting that our study shows that reported depression and anxiety of migrant children differs according to grade, which is in line with previous findings among Chinese migrant children (5), suggesting younger children reported more psychological and behavioral problems. Girls reported less depression than boys, suggesting that migrant girls adjusted better than boys in the current study, which is contradict to the general pattern of gender among young population. Yet, this result is consistent with early findings that migrant girls exhibited better school adjustment (40), social-cultural adjustment (38), and less loneliness (8). One possible explanation is that the traditional ideal of preference for boys is much more salient in rural areas. Thus, when girls moved to cities with their parents, they can enjoy more rights and access to more resource than they did in their original homes (41). Considering that rural-to-urban migrant boys outnumber girls (2), schools should pay particular attention to the mental health of migrant boys and help them to adjust to the school and urban culture.

The results of this study hold important implications for further research on the length of residence, socioeconomic, and rural-to-urban migrants’ health, as well as for implementing policy. First, this study provided evidence of a quadratic relationship between mental health and length of residence which indicates the process of psychological adjustment and changes with different stages among rural-to-urban migrant children. This is critical to deeper understandings of the complex and dynamic process of psychological adjustment. Second, the results also demonstrated that high family SES contributes to quick adaptation when moving to a new city, while low SES is a disadvantage that may lead to a prolonged low level of mental health. Mental health in adolescence has been shown to strongly influence the mental health status of young adulthood (42). These findings have implications for establishing efficient policies for providing job opportunities, raising unemployment benefits, and improving the minimum living allowance for migrant families, which in turn can improve the family’s SES. Third, it is very important to know where they moved from. It is different between from Shanghai and from a rural area. In China, most migrant children from rural areas would went the migrant school. While if migrant children came from Shanghai or other developed city, their parents could support them to other better school not migrant schools, such as international schools. In our study, the participants were from migrant schools who moved from rural areas. In future studies, the difference should be considered. Fourth, psychological guidance is very important for the elder children in migrant school.

Lastly, several limitations of the study should be considered when we interpret the findings. The arrival age, which was not assessed in this study, may influence the adaptation process. Compared to early-comers, the late comers may have more difficulties in integrating to the new environment by the same age. Study has shown that migrant children who begin attending schools in the new area before the third grade do not differ in their grades, whereas those beginning a new school later than fourth grade do not converge with native peers (43). Thus, further research should control for the age of arrival in the new environment. Second, we assessed family SES only by using the parents’ education and self-rated family economic conditions, which may not represent objectively the overall family SES. Though we asked children to report their family income in the questionnaire, the data were inaccurate and had many missing values. Third, we did not consider more variates in this study design, such as parental relationship, social support. It will be much more rigorous in future investigations.

## Conclusion

The current research tested the influence of length of residence and SES on negative mental health outcomes among migrant school students in Beijing, China. Quadratic relationships between length of residence and state/trait anxiety and depression were found. Additionally, family SES was found to play an important role in the psychological adaptation of migrant children: migrant children with lower family SES reported higher levels of anxiety and depression. Finally, length of residence interacted remarkably with SES on anxiety: trait anxiety level began to increase sooner for children with lower family SES. In sum, our study has provided evidence that mental health of migrant children in migrant schools may improve a little during the first few years but begin to decline years after having lived in cities and get worse as the length of residence increase; migrant children from low socioeconomic background are at a particular disadvantage of poorer mental health and sooner and greater deterioration. The result calls for more attention to this group and long-term intervention.

## Ethical Approval

The study design and procedure were proved by the Ethics Review Committee of the authors’ institute.

## Ethics Statement

This study was carried out in accordance with the recommendations of the Ethics Review Committee of CAS Institute of psychology with written informed consent from all subjects. All subjects gave written informed consent in accordance with the Declaration of Helsinki. Because of the survey conducted in school, their legal guardians were their headmasters and teachers. The protocol was approved by the students’ headmasters and teachers. Their parents were informed by the school about their children participating the study. It will cause no any loss or benefit of the headmasters and teachers whether the students participate our study or not. The study was approved by the Ethics Review Committee of CAS Institute of psychology.

## Author Contributions

ZL is the guarantor of integrity of entire study. He did the design and concepts of the study. He gave all kinds of supports for other authors, including ideas, method, and manuscript preparation and revising. RW, XY, and JC did questionnaire design, data acquisition, data analysis, and so on. LF, JC, and XY mainly did the data analysis, searching literatures, and writing the manuscript. RW was mainly responsible for the revision of paper and interpretation of data and support for others. JC and LF were mainly responsible for data analysis, manuscript editing, literatures searching, and submitting the paper.

## Conflict of Interest Statement

The authors declare that the research was conducted in the absence of any commercial or financial relationships that could be construed as a potential conflict of interest. The reviewer, PH, and the handling editor declared their shared affiliation, and the handling editor states that the process nevertheless met the standards of a fair and objective review.
